# Targeting the potassium ion channel genes *SK* and *SH* as a novel approach for control of insect pests: efficacy and biosafety

**DOI:** 10.1002/ps.5516

**Published:** 2019-07-29

**Authors:** Baida Alshukri, Federica Astarita, Mushtaq Al‐Esawy, Hesham Mohamed El Sayed Abd El Halim, Francesco Pennacchio, Angharad Margaret Roscoe Gatehouse, Martin Gethin Edwards

**Affiliations:** ^1^ School of Natural and Environmental Sciences, Newcastle University Newcastle‐upon‐Tyne UK; ^2^ Department of Agricultural Sciences, Laboratory of Entomology “E. Tremblay” University of Napoli “Federico II” Portici Italy; ^3^ Institute of Neuroscience, Newcastle University Newcastle‐upon‐Tyne UK; ^4^ Department of Plant Protection University of Kufa Iraq; ^5^ Entomology Department, Faculty of Science Benha University Benha Egypt

**Keywords:** insect control, RNAi, potassium ion channels, biosafety, *Tribolium castaneum*, *Apis mellifera*

## Abstract

**BACKGROUND:**

Potassium ion channels play a critical role in the generation of electrical signals and thus provide potential targets for control of insect pests by RNA interference.

**RESULTS:**

Genes encoding the small conductance calcium‐activated potassium channel (*SK*) and the voltage‐gated potassium channel (*SH*) were knocked down in *Tribolium castaneum* by injection and oral delivery of dsRNA (dsTcSK and dsTcSH, respectively). Irrespective of the delivery mechanism a dose‐dependent effect was observed for knockdown (KD) of gene expression and insect mortality for both genes. Larvae fed a 400 ng dsRNA mg^–1^ diet showed significant gene (*P* < 0.05) knockdown (98% and 83%) for *SK* and *SH*, respectively, with corresponding mortalities of 100% and 98% after 7 days. When injected (248.4 ng larva^–1^), gene KD was 99% and 98% for *SK* and *SH*, causing 100% and 73.4% mortality, respectively. All developmental stages tested (larvae, early‐ and late‐stage pupae and adults) showed an RNAi‐sensitive response for both genes. LC50 values were lower for *SK* than *SH*, irrespective of delivery method, demonstrating that the knockdown of *SK* had a greater effect on larval mortality. Biosafety studies using adult honeybee *Apis mellifera* showed that there were no significant differences either in expression levels or mortality of honeybees orally dosed with dsTcSK and dsTcSH compared to control‐fed bees. Similarly, there was no significant difference in the titre of deformed wing virus, used as a measure of immune suppression, between experimental and control bees.

**CONCLUSION:**

This study demonstrates the potential of using RNAi targeting neural receptors as a technology for the control of *T. castaneum*. © 2019 The Authors. *Pest Management Science* published by John Wiley & Sons Ltd on behalf of Society of Chemical Industry.

## 1 INTRODUCTION

Insects cause severe crop damage resulting in significant economic losses and diminished food resources at a time when the population of the world is increasing rapidly, estimated by the United Nations Population Fund to reach 9.0 billion by 2043.^1^ To achieve the goal of feeding this ever‐increasing global population, agricultural productivity has to increase by approximately 70% (FAO, 2009) requiring a paradigm shift in current thinking and practices, including that of crop protection. Thus, it is essential that new, sustainable pest control strategies be developed.

Gene silencing through RNA interference (RNAi), via the specific post‐transcriptional down‐regulation of gene expression,^2^ has been proposed as an alternative approach to mitigate the impact of insect pests. This technology can be exploited for crop protection by targeting specific essential genes and is considered to represent a highly specific means of crop protection^2^ that can be induced through an *in vivo* application of dsRNA molecules, which are homologous to the target gene. This biological process results in the degradation of a target mRNA,^3^ enabling the analysis of loss of function in organisms in which classical genetic analysis is not feasible. This technology can be seen as an ‘environmentally friendly’ approach for the control of insect pests, with a high degree of specificity.^4^ Many insect species from different orders, including Coleoptera,^5^, ^6^, ^7^ Hemiptera,^8^ Orthoptera,^9^ Diptera^5^, ^10^, ^11^ and Lepidoptera,^12^, ^13^, ^14^, ^15^ have been shown to be sensitive to dsRNA, although some species are far more sensitive than others. Previous studies have revealed that the Coleoptera are more susceptible to dsRNA compared to other arthropod orders,^16^ while the Lepidoptera require higher concentrations of dsRNA.^17^, ^18^ For example, injection at a concentration of 3 μg μL^–l^ of dsASTC or dsASTCC was required for the successful suppression of the corresponding genes in *Clostera anastomosis*,^19^ whilst only 500 ng μL^–l^ of ds*Tc*‐*ff* was required for effective knockdown of this orphan gene in *T. castaneum*.^20^ The ease of RNAi application in the red flour beetle *T. castaneum* has thus made this species a powerful screening platform for RNAi,^21^, ^22^ which can be delivered via subcuticular injection and feeding by artificial diet. *Tribolium* has a robust systematic RNAi response through its development, which makes it possible to perform RNAi at the post‐embryonic stage by injecting dsRNA into the larval body cavity.^23^ As such, RNAi phenotypes in *Tribolium* are easy to obtain and are highly reproducible; virtually all *Tribolium* tissues can respond to extracellular dsRNA, and all life stages have been induced by RNAi.^22^, ^24^

*T. castaneum* is a major insect pest of stored grain.^24^, ^25^ The antioxidant system of *T. castaneum* provides resistance to several insecticides and allows rapid adaptation to extreme temperatures, periods of drought and prolonged periods of fasting.^26^ Whilst chemical pesticides are still the major approach used to control this and other agricultural insect pests, they are associated with significant hazards to the environment, human health and non‐target insects (WHO, 2010). Furthermore, many have become resistant to these synthetic chemistries, with *T. castaneum* having become resistant to the fumigant phosphine, which is used extensively worldwide.^27^

Current insecticides most commonly target the insect nervous system, often targeting the ion channels responsible for perpetuating the action potential along neurons and the enzymes of the synaptic cleft responsible for the degradation of neurotransmitters. Recent studies have shown that targeting the voltage‐gated sodium channels, a primary target for pyrethroids, using RNAi caused approximately 80% adult mortality of *T. castaneum*.^28^ The potassium ion channels also represent viable targets for RNAi. They are composed of two parts: the filter, which allows potassium ions to pass, and the gate, which opens and closes the channel depending on environmental signals.^29^, ^30^, ^31^ These channels are involved in setting and resetting the resting potential in excitable nervous cells.^32^ The SK gene codes for small conductance calcium‐activated potassium channels, which control the action potential discharge frequency and are involved in synaptic plasticity, therefore playing important roles in the learning and memory in insects such as *Drosophila*.^33^ Meanwhile, the SH gene codes for voltage‐gated potassium channels, which are integral membrane proteins essential for the correct functioning and repolarization of the cell and *SH* helps determine the amount of sleep required by an organism, point mutations in the SH gene result in Drosophila that are not impaired by sleep deprivation.^34^, ^35^ Both genes are expressed in the central nervous system (CNS) in Coloptera.

A given pest control strategy not only has to be effective, but safe for non‐target organisms and in particular beneficial insects. The honeybee *Apis mellifera* is an essential pollinator of approximately 30% of all vegetables and fruits,^36^ with bee pollination representing a global economic worth in the region of $215 billion in food production.^37^ Unfortunately, the abundance of insect pollinator populations has declined in recent years, particularly *A. mellifera*,^38^ with one of the main causes for colony loss attributed to pesticide exposure. Compared to many other insects, honeybees are highly sensitive to pesticides and this is thought to be due to the lack of genes encoding detoxification enzymes, including cytochrome P450 monooxygenases (P450s), glutathione‐*S*‐transferases^39^ and carboxylesterases.^40^

Systemic insecticides are of particular concern to bees because they can be translocated to pollen and nectar.^41^, ^42^ The forager bees are particularly vulnerable to exposure to pesticide residues in pollen and nectar. Furthermore, they can transport the contaminated food source back to the colony, which is then fed to other castes such as larvae and the queen.^43^ Sublethal doses of pesticides can have various other effects on the honeybee's life cycle. For example, feeding honeybee larvae on pollen contaminated with chlorpyrifos reduces the emergence of queen bees.^21^ To protect insect pollinators from insecticides, the European Union has banned the use of three neonicotinoid compounds, clothianidin, imidacloprid and thiamethoxam, all of which are thought to affect bee behaviour and survival. Because of their importance both to agriculture and the natural environment, the non‐target effects of new insecticidal molecules have to be tested with honeybees as part of the registration process.

Whilst many insect species representing agricultural pests from different orders are known to be sensitive to dsRNA, several studies indicate that the RNAi machinery is present in the different developmental stages of the honeybee,^44^, ^45^ including the two Dicer enzymes and the RNA‐induced silencing complex proteins.^46^ RNAi has been applied successfully in both adult bees and larval bees in gene function analyses.^47^, ^48^ This finding makes it essential that RNAi‐based technologies for crop protection are screened against this important pollinator for potential non‐target effects.

Here we demonstrate the potential of using RNAi targeting the potassium ion channel genes, *SK* and *SH*, as a novel and effective approach to the control of *T. castaneum*, this approach resulted in significant gene knockdown and significant mortality. Furthermore, we demonstrate that *TcSK* and *TcSH* do not affect expression of the corresponding genes in bee nor affect mortality; we also show that these bees were not immuno‐compromised, as measured by the titre of deformed wing virus (DWV).

## 2 MATERIAL AND METHODS

### 2.1 Insects

A culture of *T. castaneum* was obtained from Blades Biological Ltd (Edenbridge, Kent TN8 7DX) and maintained on organic whole flour containing 5% brewer's yeast at 30 °C, 16:8 h (L:D). The flour was replaced every 2–4 weeks. Honeybees, *A. mellifera*, were obtained from the Tyneside Beekeepers Association (Newcastle upon Tyne, UK) and maintained on 50% (w/v) sucrose solution.

### 2.2 Design and synthesis of dsRNA

The sequences of the *T. castaneum* potassium ion channel *SK* and *SH* genes were identified using a *T. castaneum* small conductance calcium‐activated potassium channel protein in a BLASTn search (https://blast.ncbi.nlm.nih.gov/Blast.cgi) for the *SK* gene (gene bank accession number XM‐008195295.1) and the *T. castaneum* potassium voltage‐gated channel protein shaker for the SH gene (gene bank accession number XM‐008192853.1) at The National Center for Biotechnology Information (https://www.ncbi.nlm.nih.gov). The E‐RNAi web tool (http://www.dkfz.de/signaling/e‐rnai3//) selected a region of XM‐008195295.1 for the *SK* gene and XM‐008192853.1 for the SH gene. These transcripts had no similarity to other transcript regions in the *T. castaneum* genome. The target sequence for the kanamycin resistance gene (*Kana*), accession number JN638547, was used as a control.^49^

Sequences for the corresponding genes in *A. mellifera* were identified as above for *SK* (gene bank accession number XM‐016914844) and *SH* (gene bank accession number XM‐016914894). The primer sequences for β‐actin (used as a housekeeping gene) and DWV are described elsewhere.^50^ The specific primers were designed using NCBI/Primer‐BLAST software.

#### 2.2.1 *RNA extraction and cDNA synthesis*

Total RNA was extracted from sixth larval instar insects using a TRIzol® Plus RNA Purification Kit (Ambion) following the manufacturer's instructions. The integrity and size of total RNA isolates were investigated using 2% agarose gel electrophoresis as described in. RNA was quantified on a Nano Drop spectrophotometer (model ND‐1000, Lablech). 1000 ng of RNA was converted to cDNA for each reaction using SuperScript® II Reverse Transcriptase (Invitrogen) following the manufacturer's instructions to initiate subsequent PCR reactions.

The synthesized cDNA served as a template for PCR. Specific primers were designed using NCBI/Primer‐BLAST software and used at a final concentration of 10 μM. The primers were designed to amplify the PCR products of 181and 150 bp for *SK* and *SH*, respectively (Fig. S1; sequence of *SK* and *SH*). The reaction contained 25 μL of PCR Master Mix, 1 μL each of Forward and Reverse Primers, 1 μL of template DNA, and the final volume made up to 50 μL by adding Ambion Nuclease‐Free Water (Ambion). After gentle vortexing samples were placed in a thermal cycler (GeneAmp PCR system 9700, Applied Biosystems) as follows: 95 °C for 3 min, 35 cycles of 95 °C for 30 s, annealing at 57 °C for 30 s, first extension (*SK*, 72 °C for 11 s; *SH* for 9 s) with a final extension step of 72 °C for 10 min. Following electrophoresis, bands in the gel were purified using a QIAquick MinElute Gel Extraction kit (Qiagen) following the manufacturer's instructions and cloned into StrataClone vector pSC‐A‐amp/kan (Stratagene) following the manufacturer's instructions. The QIAprep Spin Miniprep Kit (Qiagen) protocol was used to purify the plasmid DNA. These plasmids were sent for sequencing to confirm the cloned insert.

#### 2.2.2 *Reverse transcription‐quantitative PCR*

Gene expression was evaluated via reverse transcription‐quantitative PCR (RT‐qPCR) using SYBR Green (Bioline) following the manufacturer's instructions. The regions to which primer pairs for RT‐qPCR were designed were distinct from those targeted by the dsRNA. RT‐qPCR conditions were as follows: 95 °C for 5 min, followed by 40 cycles of 15 s at 95 °C, 30 s at 57 °C and 15 s at 60 °C. Three biological replicates of cDNA as described in section 2.3.3 containing five pooled insects for each were used and normalized against a reference gene *TcRpS6* (gene bank accession number XP_968395.1). Relative transcript quantity was calculated using the ΔΔCq method.^51^

Transcription levels of *SK* and *SH* and DWV genome copies in adult honeybee were determined as above. β‐actin was used as a reference gene. The total RNA isolated from honeybees showed that >95% of honeybee colonies were infected with DWV. The standard curve was obtained by plotting the logarithm of eight 10‐fold dilutions of a starting solution containing 21.9 ng plasmid DNA using a Strata Clone PCR cloning kit (Agilent Technologies) with a DWV insert (from 21.9 ng to 2.19 fg), against the corresponding Cq values as the average of three repetitions.^50^ The relative transcript quantity of the DWV gene from honeybees fed on dsRNA targeting *T. castaneum SK* and *SH* was calculated by plotting Cq values on the standard curve using the following equation:^52^

number of copies = [amount × (6.022 × 10^23^)]/[length × (1 × 10^9^) × 650].

#### 2.2.3 *Synthesis of dsRNA*

dsRNA was synthesized using the MEGA Script T7 Kit (Ambion) following the manufacturer's instructions: 1 μg of PCR product was mixed with 10X T7 reaction buffer, T7 enzyme mix and the four ribonucleotide solutions (ATP, CTP, GTP, and UTP), and incubated at 37 °C overnight, followed by 75 °C for 5 min. The dsRNA was bound to filter cartridges. Eluted dsRNA was stored at −80 °C and quantified prior to use.

### 2.3 Stability of dsRNA in sucrose solution

The stability of dsRNA in sucrose solution was evaluated by incubating 1 μg of dsRNA for *SH* and *SK* in 10 μL of 50% (w/v) sucrose solution at 34 °C^5^ at the following time points: 0, 6, 12, 18, 24, and 48 h. The integrity of the dsRNA was analysed by separation on 2% (w/v) agarose gels, and bands visualized by ethidium bromide staining under UV.

### 2.4 Delivery of dsRNA by injection

*T. castaneum* adults, pupae and larvae were injected using a NanojectII™ injector (Drummond Scientific Company) under a dissecting stereomicroscope. Sixth larval stage insects were injected into the dorsal side between the first and second abdominal segments with dsRNA at a range of doses (62.1, 124.2, 186.3 and 248.4 ng larva^–1^); pupae were injected with dsRNA (248.4 ng pupa^–1^) between the second and third abdominal segments.^23^ Adults were injected on the dorsal side under the elytron (248.4 ng adult^–1^). Injected insects were left for 15 min and then transferred to Petri dishes containing white flour supplemented with brewer's yeast (5% w/w) we at 30 °C. Three biological replicates were used. Each replicate consisted of 15 insects for the survival study and five insects for RT‐qPCR. Expression of targeted genes was quantified 48 h post exposure to dsRNA. Survival was monitored on a daily basis for 7 days. For all injection assays, three different controls were used: insects without injection (control 1), insects injected with RNAase‐free water (control 2) and insects injected with dsRNA kanamycin at 248.4 ng insect^–1^ (dsKana), targeting a region of the bacterial resistance gene (control 3).

### 2.5 Delivery of dsRNA by feeding

#### 2.5.1 T. castaneum

The dsRNA was delivered via flour disks prepared (Xie *et al*. 1996). 10 μL of flour suspension (dsRNA, 5% brewer's yeast) was prepared in flat‐bottomed wells of a 96‐well microtiter plate and allowed to dry at room temperature and an individual third instar larva was placed in each well. All insects were fed for 72 h on flour disks at a range of concentrations (100, 200, 300, 400 ng dsRNA mg^–1^ diet). Three groups of control were used: flour only (control 1), flour with RNAase‐free water (control 2) and flour with RNAase‐free water + dsKana (200 ng dsKana mg^–1^ diet) (control 3). The diet was changed every 2 days to prevent contamination and degradation of dsRNA. Three biological replicates were carried out. Each replicate consisted of 15 insects for the survival study and five insects for gene expression studies.

#### 2.5.2 A. mellifera

For survival studies, four treatments were used, each with five biological replicates of ten foragers. Bees were fed daily with 2 mL of diet as follows: treatment 1, 50% (w/v) sucrose solution containing 20 ng uL^–1^ *T. castaneum* dsSK (dsTcSK); treatment 2, 50% (w/v) sucrose solution containing 20 ng uL^–1^ *T. castaneum* dsSH (dsTcSH); control 1, 50% (w/v) sucrose solution; control 2, 50% (w/v) sucrose solution containing bacterial dsKana. Bees were reared in an incubator at 34 °C and 75–80% relative humidity, 24 h dark. Survival was monitored daily for 10 days. Bees clearly attracted and consumed dsRNA solution as it was mixed with the sucrose solution compared to water consumption (Fig. 3.1). For survival studies, *n* = 50 per treatment (five biological replicates of 10 foragers); for gene expression studies by RT‐qPCR, *n* = 25 per treatment.

### 2.6 Statistical analysis

Insect mortality was analysed using Kaplan–Meier survival analysis and the Sigma Plot program (version 12.5, Systat. Software Inc., San Jose, USA), and insect mortality was corrected according to Abbott's formula.^53^ RT‐qPCR results were analysed with one‐way ANOVA followed by the Tukey test to compare differences in the effect of various concentrations of dsRNA (Minitab, State College, PA, USA).

## 3 RESULTS

### 3.1 Synthesis of dsRNA

Sequence alignment confirmed 91% and 95% homology between the insert and the coding sequence of *SH* and *SK*, respectively (Fig. S1). Quantification demonstrated that it was feasible to produce sufficient quantities of synthetic dsRNA for subsequent studies, these being 5000 ng μL^–1^, 3456 ng μL^–1^ and 1500 ng μL^–1^ for dsSK, dsSH and dsKana. Migration of dsRNA was slower than for the corresponding DNA fragment of the same length due to the diameter of dsRNA, which is 30% larger than that of DNA.^54^

### 3.2 Endogenous expression of *SH* and *SK* in *T. castaneum*

**Figure 1:**
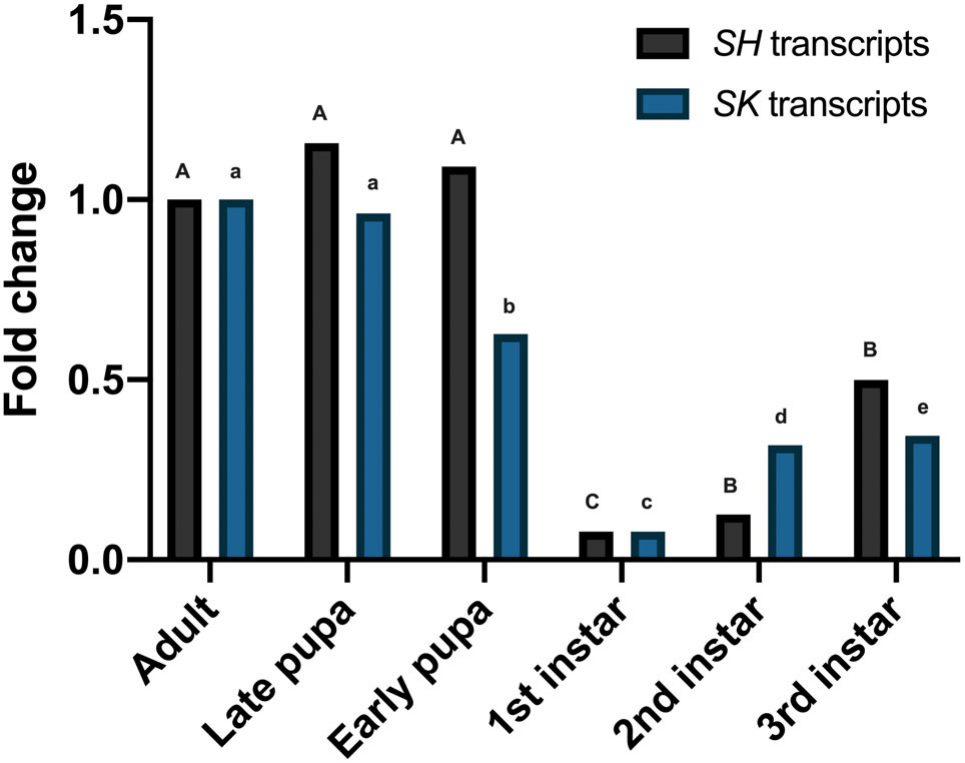
Stage‐specific expression of *SK* and *SH* in the whole body of *T. castaneum* determined by RTqPCR. *n* = 5; mean of replications is shown. Means with different letters are significantly different (*P* < 0.05, one‐way ANOVA, post‐hoc Tukey test) within each gene set. Expression levels are normalized against the *Tc*RpS6 gene as an internal standard.

Relative abundance of the *TcSK* and *TcSH* at different developmental stages of *T. castaneum* was investigated by RT‐qPCR to ensure that subsequent studies were carried out on appropriate developmental stages; TcRpS6 was used as a reference gene for gene expression. Both *SK* and *SH* were shown to be expressed in all developmental stages (first, third and sixth instar larvae; early‐ and late‐stage pupae and adults). Expression of *SK* and *SH* in late pupae was 0.91 and 1.1‐fold, respectively, relative to the adult stage, but these small differences were not significant (*P* > 0.05; Fig. 1). Expression of *SK* and *SH* in the early pupal stage was 0.6 and 1‐fold respectively, and whilst these differences were significant between early and late pupae for *SK*, they were not significant for *SH*. The expression of both genes was much lower in the larval stages (first, third and sixth) compared to either adults or pupae, being 0.07, 0.11 and 0.46‐fold for *SH* and 0.07, 0.30, 0.34‐fold change for *SK* (*P* < 0.05). These results suggest that apart from the early larval instars, most developmental stages are viable targets for RNAi KD of potassium ion channel genes.

### 3.3 Delivery of dsRNA on expression of target genes in *T. castaneum*

#### 3.3.1 *Delivery via injection*

**Figure 2:**
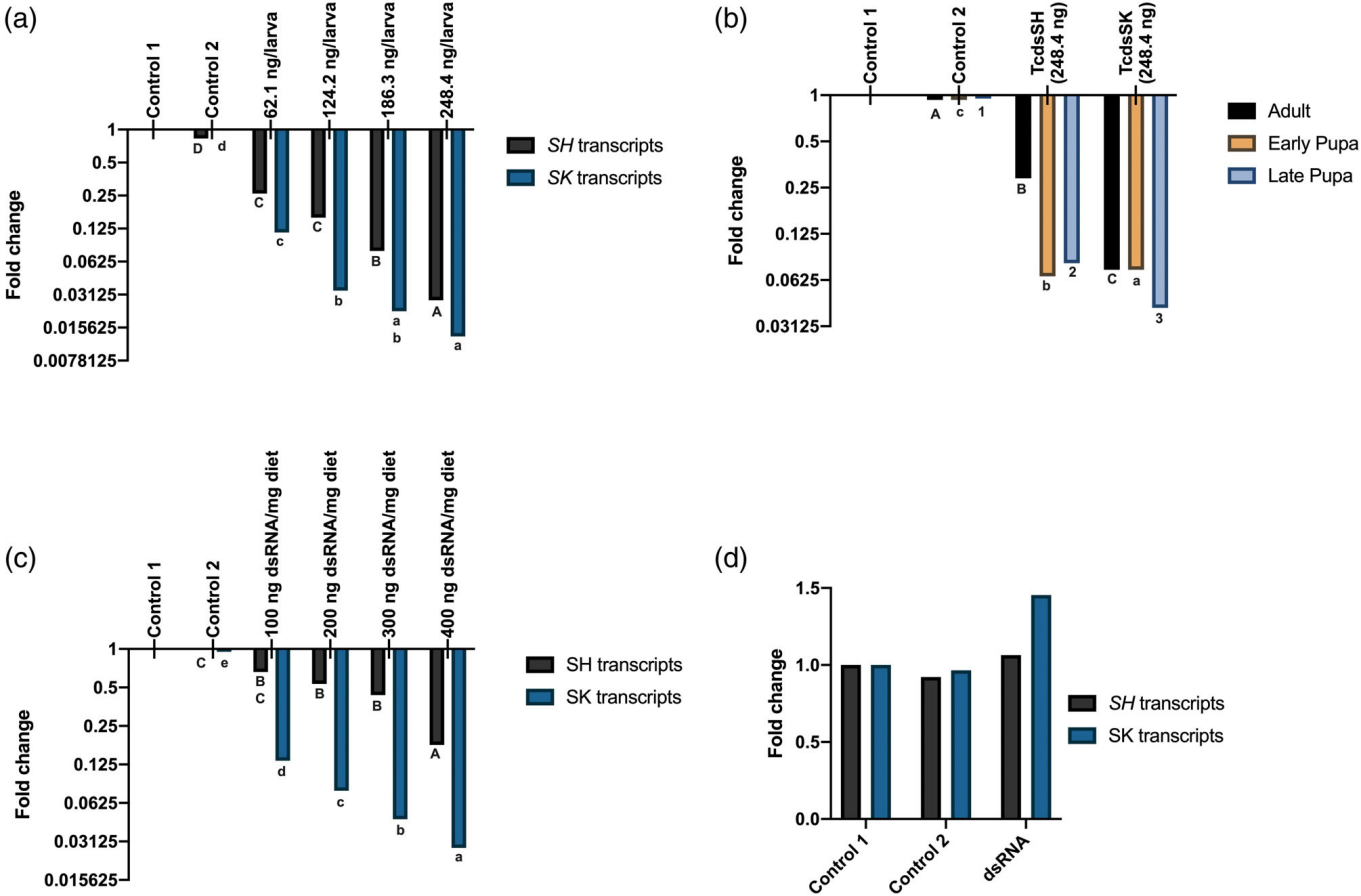
(a) Expression of *SK* and *SH* in larvae injected with dsRNA after 48 h. Control 1, larvae injected with RNAase free water; control 2, larvae injected with dsKana. Expression levels were normalized against the T*Tc*RpS6 gene as an internal standard. *n* = 5; mean of replications is shown. Means with different letters are significantly different (*P* < 0.05, one‐way ANOVA, post‐hoc Tukey test) within each gene set. (b) Expression of SK and SH in early‐ and late‐stage pupae and adults injected with dsRNA after 48 h. Control 1, larvae injected with RNAase free water; control 2, larvae injected with dsKana. Expression levels were normalized against the *Tc*RpS6 gene as an internal standard. *n* = 5; mean of replications is shown. Means with different letters are significantly different (*P* < 0.05, one‐way ANOVA, post‐hoc Tukey test) within each gene set.(c) Expression of SK and SH in larvae fed with dsRNA after 72 h. Control 1, larvae injected with RNAase free water; control 2, larvae injected with dsKana. Expression levels were normalized against the *Tc*RpS6 gene as an internal standard. *n* = 5; mean of replications is shown. Means with different letters are significantly different (*P* < 0.05, one‐way ANOVA, post‐hoc Tukey test) within each gene set. (d) Expression of SK and SH in foraging honeybees *A. mellifera* fed *Tc*dsRNA after 72 h. Treatments, foragers fed 50% sucrose containing 20 ng μL^−1^ *Tc*dsSK or *Tc*dsSH; control 1, foragers fed 50% sucrose; control 2, foragers fed 50% sucrose containing 20 ng μL^−1^ bacterial dsKana,. Expression levels were normalised against the β‐actin gene as an internal standard. Mean of replications are shown. Means with different letters are significantly different in the expression levels of the target gene (*P* < 0.05, one‐way ANOVA, post‐hoc Tukey test).

Injection of dsRNA into sixth instar larvae showed a dose–response effect on gene knockdown 48 h post treatment, with injections at 62.1, 124.2, 186.3 and 248.4 ng larva^–1^ reducing expression of *SK* by 0.11‐, 0.03‐, 0.02‐ and 0.01‐fold, respectively; expression of *SH* was reduced 0.26‐, 0.15‐, 0.07‐ and 0.027‐fold at these same concentrations (Fig. 2). In contrast, no significant (*P* > 0.05) KD in *SK* or *SH* expression was observed for either control 1 (RNAase free water) or control 2 (248.4 ng larva^–1^ Kana dsRNA). These results indicate that RNAi can cause almost complete knockdown in the expression of these two target genes in final instar larvae.

RT‐qPCR analyses of mRNA transcript levels showed that early‐ and late‐stage pupae and adults were all sensitive to the effects of RNAi, resulting in reduced relative expression of both *SK* and *SH* post injection (248.4 ng pupa^–1^ of dsRNA). As observed for larvae, greater KD of *SK* occurred in both the late pupae and adults compared to that observed for *SH*, although no differences in KD of expression were observed between these two genes in the early pupal stages, suggesting that early‐stage pupae were equally sensitive. As expected, no reduction in expression of either *SK* or *SH* was observed in the controls (Fig. 2(b)). These findings show that dsRNA led to a significant reduction in levels of the mRNA of the target genes 48 h post injection in all developmental stages investigated.

#### 3.3.2 *Oral delivery*

Three days of continuous feeding of third instar larvae on flour disks containing the dsRNA at a range of different concentrations (100, 200, 300 and 400 ng dsRNA mg^–1^ diet) caused a dose–response decrease in *SK* (0.13‐, 0.07‐, 0.04‐ and 0.02‐fold) and *SH* (0.66‐, 0.53‐, 0.43‐ and 0.17‐fold) transcript levels; again no significant decrease was observed in either of the controls (Fig. 2(c)). Also, as observed for the injection studies, there was greater down‐regulation of expression of the *SK* (reaching a maximum of 98%) compared to *SH* (83%).

### 3.4 Delivery of dsRNA targeting potassium ion channel genes on survival of *T. castaneum*

#### 3.4.1 *Delivery via injection*

**Figure 3:**
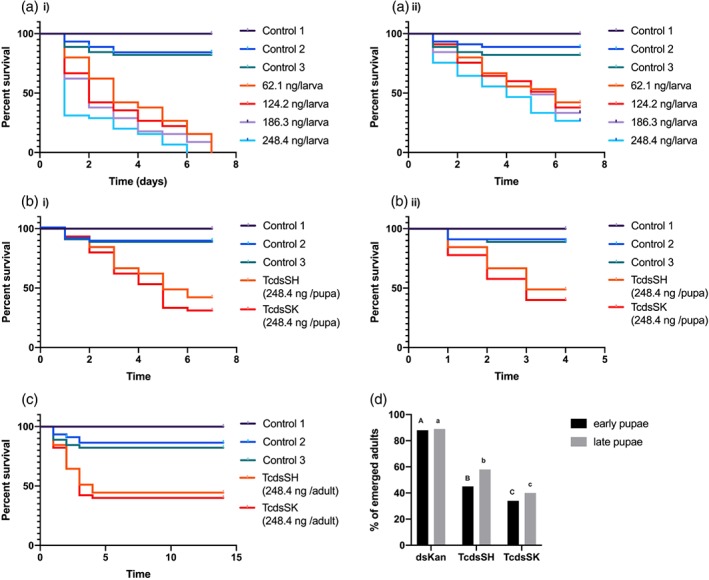
(a) Survival of *T. castaneum* larvae injected with dsRNA targeted to (a) *SK* and (b) *SH*. Control 1, untreated larvae; control 2, larvae injected with RNAase free water; control 3, larvae injected with dsKana. *n* = 45 larva/treatment, *P* < 0.001, by Kaplan–Meier survival analysis. (b) Survival of *T. castaneum* (a) early‐ and (b) late‐stage pupae injected with dsSK or dsSH. Control 1, untreated pupae; control 2, pupae injected with RNAase free water; control 3, pupae injected with dsKana. *n* = 45 pupa/treatment; *P* < 0.001, by Kaplan–Meier survival analysis. (c) Survival of *T. castaneum* adults injected with dsSK or dsSH. Control 1, untreated pupae; control 2, pupae injected with RNAase free water; control 3, pupae injected with dsKana. *n* = 45 pupa/treatment; *P* < 0.001, by Kaplan–Meier survival analysis. (d) Percentage emergence of adult *T. castaneum* from early‐ or late‐stage pupae post injection. Mean ± SD of three replications is shown. Means with different letters are significantly different (*P* < 0.05, one‐way ANOVA, post‐hoc Tukey test) within each gene set.

Larval survival was significantly (*P* < 0.001) reduced in a dose‐dependent manner post injection with either dsSK or dsSH compared to the three different control groups. No mortality was observed during the 7‐day trial period in control 1. The mortalities recorded in control groups 2 and 3 were 11.2% and 17.8% respectively, but were not significantly different (*P* > 0.001) to those for group 1. After 7 days 100% mortality was recorded for all concentrations of dsSK (Fig. 3). However, after this same time period dsSH caused 57.8%, 62.3%, 66.7% and 73.4% mortality, respectively, for the increasing concentrations of administered (62.1, 124.2, 186.3 and 248.4 ng larva^–1^). The LC_50_ values of the larvae injected with dsSK and dsSH were 2.38 and 34.93 ng larva^–1^, respectively, on day 6 (Table 1), reflecting the relative levels in knockdown in gene expression (see above).

**Table 1:** LC_50_ of SK and SH dsRNA towards *T. castaneum* delivered by both oral and injection assays Injection LC_50_ recorded and the injected dose of dsRNA per larva, oral LC_50_ is recoded as the concentration of dsRNA in the flour disc diet bioassay.

Delivery	SK dsRNA	SH dsRNA
Injection	2.38 ng larva^–1^	34.93 ng larva^–1^
Oral	65 ng mg^–1^	117.01 ng mg^–1^

The mortalities of both early‐stage, measured 7 days post injection (248.4 ng pupa^–1^), and late‐stage pupae (4 days post injection) for both dsSK and dsSH were significantly different (*P* < 0.001) to those of the three control groups. No mortality occurred in control 1 for either early‐ or late‐stage pupae, whilst mortalities for controls 2 and 3 were 9.1% and 11.5%, respectively, for early pupae, and 11.1% for both these control groups for late‐stage pupae, but these differences among the three control groups were not significant (*P* > 0.001). When injected, dsSK caused 68.9% and 57.8% mortalities for the early‐stage and late‐stage pupae, whilst dsSH resulted in mortalities of 60% and 51.2% for early‐stage and late‐stage pupae (Fig. 3(b)). These results reflect the trends reported for larvae, which were also more sensitive to dsSK compared to dsSH. The mortality figures were reflected in reduced adult emergence irrespective of whether the early‐ or late‐stage pupae had been treated (Fig. 3(d)). Percentage emergence was significantly lower in the dsSK group compared to the dsSH group, which in turn was significantly different to the controls, even those injected with dsKana.

The mortalities of adults injected with either dsSK or dsSH (248.4 ng adult^–1^) after 2 weeks were 60.0% and 55.55%, respectively, compared to control 1, where again no mortality occurred during this period (Fig. 3(c)). As seen with pupae, low, but not significant (*P* < 0.001), mortality occurred for control groups 2 and 3, at 13.3% and 17.3%, respectively.

#### 3.4.2 *Oral delivery*

**Figure 4:**
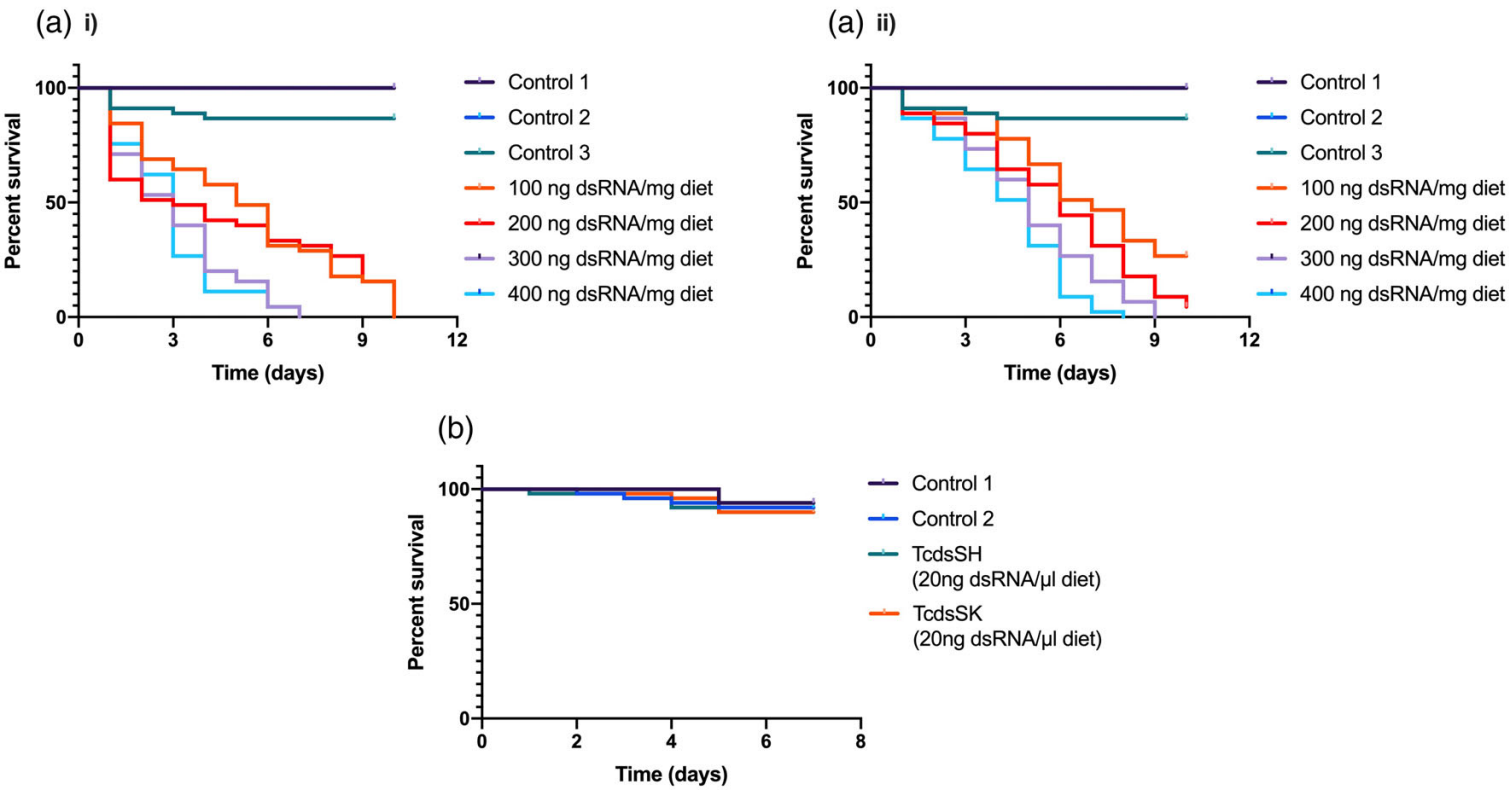
(a) Survival of *T. castaneum* larvae fed (a) dsSK or (b) dsSH. Control 1, larvae fed flour disks; control 2, larvae fed flour disks containing RNAase free water; control 3, larvae fed flour disks containing dsKana. *n* = 45 larva/treatment; *P* < 0.001, by Kaplan–Meier survival analysis. (b) Survival of *A. mellifera* foragers fed 50% sucrose containing T*Tc*dsRNA targeting *SK* or *SH*. Control 1, foragers fed 50% sucrose; control 2, foragers fed 50% sucrose containing 20 ng μL^–1^ bacterial dsKana. *P* < 0.001, by Kaplan‐ Meier survival analysis.

The induction of RNAi via oral delivery was investigated in third instar larvae at the same range of concentrations of dsRNA as used to determine the effects on gene KD (100, 200, 300 and 400 ng mg^−1^ diet). Abbott's formula was used to correct survival data relative to control 1 (untreated control). There was a clear dose–response effect for both dsSK and dsSH (Fig. 4(a),(b)). Exposure to dsSK caused 100% mortality at all concentrations tested by day 10, although the highest concentration resulted in >90% mortality by day 4. Exposure to dsSH, on the other hand, only resulted in 100% at the two higher concentrations tested, these being on days 8 and 9 for doses of 400 ng mg^–1^ diet and 300 ng mg^–1^ diet, respectively; the two lower doses only achieved mortality levels of up to 73% and 91% during the 10‐day period. The LC_50_ values for *SK* and *SH* were 65 and 117.01 ng mg^–1^, respectively, on day 6 (Table 1), again reflecting results obtained when dsRNA was delivered via injection. Meanwhile, no mortality occurred in either control 1 or 2, and there was only 13.3% mortality for control 3; these values are all significantly (*P* < 0.001) different to those for the dsRNA‐fed insects.

### 3.5 Biosafety studies with honeybee *A. mellifera* foragers

#### 3.5.1 *Bioinformatic analysis of targeted gene sequences*

MegaBLAST homology searches confirmed that there was no significant sequence similarity for the dsRNA of *T. castaneum* designed to target *SK* transcripts used in the present study to any other insect species or human full‐length sequences. In addition, restricting MegaBLAST searches to *A. mellifera* yielded no matches across the known honeybee full length sequence. However, there was a 15.2% nucleotide sequence similarity between the dsRNA of *T. castaneum* for *SH* used in the present study with that of the corresponding full‐length *SH* transcript of *A. mellifera*. Alignments between the shorter overlapping regions between the dsRNA fragments and their respective target transcript showed that the 150 bp *Tc*dsSH shared 132 bases with the *A. mellifera* transcript (88%), of which there were two regions of more than 24 contiguous bases, and the 181 bp *Tc*dsSK was homologous to 77 bases of the honeybee transcript (42%). This region did not contain any contiguous runs of sequence greater than 24 nucleotides in length (Fig. S2a,b). Furthermore, when restricting the *Tc*dsSK sequence to just contain the most homologous region towards the honeybee transcript the level of similarity was 79% (77/97) with no contiguous runs of sequence greater than 24 nucleotides (Fig. S2b). Neighbour‐joining tree analysis further demonstrated the evolutionary difference between the two dsRNA fragments and known sequences from insect orders (Fig. S2c,d).

#### 3.5.2 *Effects of* T. castaneum *dsSK and dsSH on gene expression levels in* A. mellifera

Prior to feeding studies, the stability of the dsRNA in the diet was investigated over a period of 48 h. Incubation of 1 μg of dsSK and dsSH of *T. castaneum* (*Tc*dsSK, *Tc*dsSH) with 10 μL of 50% (w/v) sucrose solution at 34 °C had little effect on the stability of the dsRNA over this time period, with only 2% degradation for the dsSH, although this was slightly higher for dsSK with 13% degradation (data not shown).

The potential for dsRNA designed to target *T. castaneum* potassium ion channel genes to knockdown the corresponding genes in honeybee (i.e. non‐target effects) was investigated in foragers after continuous feeding of either *Tc*dsSK or *Tc*dsSH (20 ng μL^–1^) for a period of 72 h. RT‐qPCR revealed that there were no significant differences in expression of either gene (*P* > 0.05) compared to the control groups over this same time period, with increases in expression of 1.06‐ and 1.4‐fold for *SK* and *SH*, respectively, relative to control 1 (Fig. 2(d)). The oral uptake of off‐target dsRNA (control 2; dsKana) also did not affect the expression of either *SK* or *SH* relative to those fed on the basic diet (control 1), with expression of *TcSK* and *TcSH* in control 2 fed honeybees being 0.92‐ and 0.96‐fold, respectively, relative to that of the control 1 group (Fig. 2(d)).

#### 3.5.3 *Effects of* T. castaneum *dsSK and dsSH on survival of* Apis mellifera

As part of the safety evaluation of the technology, honeybee foragers were fed a 50% sucrose solution containing either *Tc*dsSK or *Tc*dsSH (20 ng μL^–1^); these doses were based on those known to induce RNAi in bee by oral feeding (Maori *et al*., 2009; Liu *et al*., 2010). After 8 days, there were no significant differences (*P* > 0.001) between the mortality of honeybees fed TcdsSK or TcdsSH with either of the two control‐fed groups. Survival remained high for all four groups throughout the trial period, with 6% and 8% mortality for control 1 and control 2, and 10% and 8% mortality for TcdsSK and TcdsSH, respectively. Honeybee survival was thus unaffected by the treatment.

#### 3.5.4 *Detection of deformed wing virus in honeybee*

**Figure 5:**
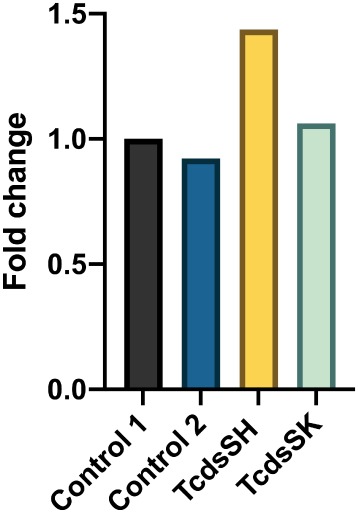
DWV titre in *A. mellifera* foragers fed *Tc*dsRNA targeting SK or SH for 72 h. Treatments, adults fed 50% sucrose containing *Tc*dsSH (20 ng μL^–1^) or *Tc*dsSK (20 ng μL^–1^). Control 1, adults fed 50% sucrose; control 2, adults fed 50% sucrose containing 20 ng μL^–1^ bacterial dsKana. Expression levels were normalised against β‐actin gene as an internal standard. Mean of replications is shown. Means were not significantly different (*P* < 0.05, one‐way ANOVA, post‐hoc Tukey test) within each gene set.

[Correction added on 30^th^ July 2019, after first online publication: The section heading has been updated in this version.] Studies were carried out to investigate the effects of TcdsRNA targeted to *SK* and *SH* on the immunocompetence of forager honeybees by evaluating levels of DWV in bees fed for 72 h on either TcdsSK or TcdsSH. RT‐qPCR analysis revealed that DWV transcript levels in foragers fed on 50% sucrose containing 20 ng μL^–1^ TcdsRNA were not significantly different (*P* > 0.05) to those of either control group, being 1.3‐ and 1.2‐fold, respectively (Fig. 5). Again there were no significant differences between the two control groups, with expression in the control 2 group being 0.97‐fold relative to control 1. The level of DWV in bees fed sucrose solution containing TcdsSK or TcdsSK (20 ng μL^–1^) was quantified and normalized to actin, with titres for DWV being 2.82 ± 0.003 × 10^4^ and 2.81 ± 0.004 × 10^4^ copies bee^–1^, respectively. Titres for control groups were 2.80 ± 0.005 × 10^4^ and 2.79 ± 0.003 × 10^4^ copies bee^–1^ for controls 1 and 2, respectively (Table 2). There was no significant difference (*P* > 0.05) between the two treatments or the group controls.

**Table 2:** Deformed wing virus titre in honeybee foragers fed on the dsSK and dsSH of *T. castaneum* and bacterial dsKana as a control

Treatment	Viral titre (copies/bee)
Control: 50% sucrose diet	2.80 × 10^4^ (±0.005)
Control: 50% sucrose diet with 20 ng μL^–1^ dsKana	2.79 × 10^4^ (±0.003)
50% sucrose diet with 20 ng μL^–1^ ds*SK*	2.82 × 10^4^ (±0.003)
50% sucrose diet with 20 ng μL^–1^ ds*SH*	2.81 × 10^4^ (±0.004)

## 4 DISCUSSION

### 4.1 Efficacy of RNAi‐based approaches

Targeting potassium ion channels using RNA interference is a novel and potentially highly exploitable way to control *T. castaneum* and other coleopteran insects. This study has demonstrated for the first time that the expression of the two genes *SK* and *SH*, which encode K^+^ channels in the nervous system, can be effectively knocked down to induce mortality.

The evolution of resistance of insect populations to synthetic insecticides poses a serious threat to crop protection, requiring the development of effective, but safe, alternative strategies. RNAi is being developed as an alternative approach,^3^ both when delivered via transgenic plants such as in SmartStaxPro, a maize hybrid expressing both Bt‐toxins and dsRNA towards Diabrotica spp. *Snf7* transcripts (Monsanto and Dow AgroSciences),^55^, ^56^ and as a biopesticide. Many current synthetic insecticides target the CNS, including ion channels. For example, pyrethroid insecticides such as permethrin, cypermethrin, fenvalerate and cyfluthrin target the voltage‐gated sodium ion channels, disrupting the normal transmission of nerve impulses.^57^ Potassium ion channels are present in most cells of eukaryotic and prokaryotic organisms, controlling a variety of cellular functions^58^ and represent another target. Targeting of the inward‐rectifying potassium channels (K_ir_) of *Aedes aegypti* by the injection of VU573, which is a synthetic organic molecule inhibiting mosquito K_ir_ channels, into the hemolymph of adult females disrupted the production and excretion of urine within 24 h, leading the authors to propose this molecule as a potential mosquitocide. Beyenbach *et al*.^59^ also confirmed that K^+^ channels represent good targets for RNAi‐based pest control strategies.

The present studies clearly demonstrate that *SK* and *SH* are highly expressed in late pupal and adult stages compared to the larval stages, and in particular compared to the early instar larvae. This result is consistent with the high levels of phenotypic differentiation observed in this insect species and the neuronal remodelling processes, which occur during metamorphosis and which are necessary for adult memory.^60^ Furthermore, transcript levels of the two genes were present in all developmental stages of this insect, but relative expression levels were different based upon the stage. The findings for the voltage‐gated potassium channel reported here are in broad agreement with those recently reported for the voltage‐gated sodium ion channel^28^ for the same species, where expression of the two genes responsible for coding these particular ion channels was highest in late‐stage pupae, followed by adults and was lowest in the larvae.

We also show that RNAi causes substantial and significant down‐regulation of the two target potassium ion channel genes *SK* and *SH*, where oral delivery of the dsRNA to third instar larvae caused up to 98% and 83% knockdown in expression after 72 h feeding at the highest concentration tested. Our results show that oral delivery of dsRNA could induce sufficient RNAi to knock down target genes, with similar efficacy to dsRNA delivered by injection. Similar levels of gene KD occurred 48 h post injection of sixth instar larvae. These data suggest that both delivery systems are equally effective. However, Laudani *et al*.^61^ found that the transcript levels for the ecdysone receptor gene were reduced to 93% following injection of a single dose of dsRNA (150 ng) whilst, when delivered orally, only 34% KD was achieved. Similarly, El Halim *et al*.^28^ found that delivery via injection was more effective than oral delivery, with 51% knockdown in expression of the sodium ion channel paralytic A gene being achieved when the dsRNA was delivered via the diet compared to 71% when injected. [Correction added on 30^th^ July 2019, after first online publication: The sentence has been updated in this version.] In keeping with these studies, Whyard *et al*.^62^ also reported successful KD of *vATPase* expression in *T. castaneum* when the dsRNA was orally delivered. However, in a more recent study, Spit *et al*.^63^ failed to observe any knockdown effects via oral delivery, despite using the same insect species. One possible explanation for this may be due to the use of different strains of *T. castaneum* causing differences in the efficiency of RNAi, as demonstrated by Swevers *et al*.,^4^ who reported that different strains of this particular insect showed different levels of susceptibility to dsRNA.

Another important finding from the present study is that down‐regulation of expression for both genes resulted in significant larval mortality, irrespective of the method of delivery of the dsRNA. Mortality is likely to be a consequence of loss of function of *SK* and *SH* causing a delay in repolarization of neurons, leading to constant firing and, eventually, neuro‐degeneration. Another likely reason for the observed mortality is impairment of K^+^ channels in the muscle, as supported by Elkins *et al*.,^64^ who demonstrated that a mutation of *Drosophila* slowpoke gene specifically abolishes a Ca^2+^‐dependent K^+^ current, significantly affecting the action potential of muscle, resulting in death. In the present study the LC_50_ values for mortality for larvae injected with *SK* and *SH* dsRNA were 2.39 and 34.93 ng larva^–1^, respectively, compared to 65 and 117.01 ng mg^–1^ diet 6 days post‐delivery by feeding. This compares with LC_50_ values of 79.89 ng larva^–1^ by injection and 150.23 ng mg^–1^ by feeding at day 6 in response to targeting the *TcNa*_*v*_ gene in *T. castaneum*.^28^ In contrast, oral delivery of dsRNA targeted against calcium channel genes via an RNAi‐based approach failed to induce mortality in *T. castaneum*.^49^ These results suggest that targeting the potassium channel genes *SK* and *SH* is greater and more effective than targeting sodium or calcium ion channel genes, at least in *T. castaneum*. Our results also indicate that *SK* and *SH* are more sensitive targets compared to other recent targets. For example, whilst targeting the acetylcholinesterase gene *TcAcel* was shown to be effective, leading to 100% larval mortality within 2 weeks following injection of the dsRNA, much higher concentrations of the dsRNA were required.^65^ Similarly, Sang *et al*.^66^ demonstrated that injection of the dsRNA (200 ng) of insulin receptor genes *T.cas‐ir1* and *T.cas‐ir2* caused 100% and 42% mortality of late instar larvae of *T. castaneum*; this stadium is comparable to the sixth instar used in the present study. [Correction added on 30^th^ July 2019, after first online publication: The sentence has been updated in this version.] The present study indicates that targeting the small conductance calcium‐activated potassium channel gene (*SK*) is more effective at inducing RNAi than targeting the voltage‐gated potassium channel gene (*SH*). These findings further support the concept that calcium‐activated potassium channels could act as significant target sites for the control of insect pests. A study with cockroaches, *Periplaneta americana*, found that the neurotoxic effect of dimethyl disulfide (DMDS) on calcium‐activated potassium channels occurred through complex regulatory pathways increasing the intracellular calcium concentration responsible for the abrogation of this channel, leading to higher toxicity.^67^

Although the use of RNAi shows great potential for control of insect pests, its application is currently limited to the control of specific insects and particularly coleopterans such as *T. castaneum*, which, in contrast to other orders, exhibit a strong RNAi response. However, the RNAi response is often reduced due to mechanisms of transporting the dsRNA from within the insect gut. The longevity of dsRNA in the midgut of the insect may be strain dependent and be subject to the action of gut nucleases on the ingested dsRNA.^63^ One reason for this strong response is that the genome of several coleopteran insects is known to include two or even three *Sid‐1*‐like genes, which are necessary for RNAi pathways.^68^ Furthermore, Cappelle *et al*.^69^ suggest that coleopterans have an additional pathway called the receptor‐mediated endocytosis pathway, which is involved in dsRNA uptake. The findings of our study provide strong evidence that potent knockdown occurred using RNAi at different developmental stages of *T. castaneum* and are consistent with the findings from other studies targeting different genes.

### 4.2 Non‐target effects

It is essential that any technology that is developed for the control of insect pests is safe and poses negligible risks to non‐target organisms and in particular those providing ecosystem services. The use of RNAi technology could, potentially, pose such a risk. For example, the consumption of sufficient quantities of dsRNA may lead to the induction of the RNAi machinery of non‐target organisms (NTOs) and the suppression of a corresponding mRNA transcript homologous to the sequence,^70^ causing loss of gene function, thus adversely impacting on the NTO.^71^ Furthermore, small doses of dsRNA can cause 90% gene knockdown in coleopterans, and the effect may stay for the long‐term and be passed on to subsequent generations.^72^ The safety of RNAi technology can be predicted only if bioinformatics data demonstrate that the dsRNA used does not show sufficient sequence similarity with non‐target species. Both Elbashir *et al*.^39^ and Tijsterman and Plasterk^73^ indicated that activation of the RNAi machinery in the organism's cells requires the introduction of dsRNA matching around 21–25 bp of mRNA. To address this important concern, we selected the honeybee *A. mellifera* since not only are they important pollinators but they are highly sensitive to dsRNA, exhibiting a strong RNAi response.^47^, ^74^

Our results clearly show that the oral delivery of dsRNA targeted to *T. castaneum SK* and *SH* genes did not result in knockdown in expression of the corresponding genes in honeybee. Importantly, there were no significant effects on survival or the immunocompetence of honeybee.

Sequence alignments indicated the presence of more than 29 nucleotides of identical regions between the dsSH from *T. castaneum* and the corresponding *SH* gene in *A. mellifera*, but no homology between the *SK* genes. However, despite 15.2% nucleotide sequence similarity for *SH*, the results show that survival of honeybees fed on sucrose solution containing dsSH (20 ng μL^–1^) was not affected and neither was there any evidence of gene knockdown. Not surprisingly, due to the lack of sequence similarity, dsSK also showed no effects. A similar observation in *A. mellifera* was reported by Powell *et al*.,^5^ who demonstrated that injections into honeybees of 50 ng of dsRNAs *Laccase 2* and vacuolar*‐ATPase V‐type subunit A*, designed to target *Aethina humidity*, similarly had no effect on bee survival and did not induce the suppression of either of the target genes. An alignment of *A. mellifera* and *A. tumida Laccase 2* and *V‐ATPase subunit A* mRNAs indicated sequence identities of 74% and 68%, respectively. Our findings are also consistent with those of other studies which have found that, although the nucleotide sequence identities between *Diabrotica virgifera* and *Leptinotarsa decemlineata* were 83% for *V‐ATPase subunit A*, there were no effects of *D. virgifera V‐ATPase subunit A* dsRNAs on the survival of *L. decemlineata*.^75^ The reason for this is not clear. However, is likely to be related to the specific segment of mRNA not shared between the target and non‐target insects, thus preventing the disruption of gene expression in the non‐target insect or a recalcitrant RNAi response in certain insects to orally delivered dsRNA molecules.

In insects there is a close association between the nervous system and the immune system.^76^ Since the target genes *SK* and *SH* play a role in the nervous system, it was important to investigate if there were more subtle effects occurring and particularly any effects of the dsRNA on the immune system of the honeybee. For these studies we analysed the titre for DWR as a measure of immunocompetency. DWV causes atrophied wings or paralysis of the legs and wings of adult honeybees and is prevalent in colonies infested with varroa mites.^77^ The varroa mite has been shown to cause amplified levels of DWV, ranging from 10% to 100%.^78^ In the absence of mites, the virus is thought to persist in bee populations as a covert infection transmitted orally between adults (nurse bees), since the virus can be detected in hypopharyngeal secretions (royal jelly) and brood food, and is transmitted vertically through the queen's ovaries and the drone's sperm. The results provided in this study demonstrate that there are no significant differences in viral titre between honeybees fed on dsSK or dsSH compared to any of the control groups. The treated and control honeybees contained approximately 2.8 × 10^4^ copies of virus per bee, whereas that of symptomatic honeybee was recorded at 3.3 × 10^10^ copies of virus per honeybee. A study by Highfield *et al*.^79^ estimated the level of DWV to fluctuate between <10^2^ and 4.2 × 10^9^ copies per asymptomatic worker. However, for symptomatic honeybees, recorded values range from 1.8 × 10^10^ to 6.9 × 10^11^ DWV per worker. Therefore, these results provide further support for the safety and use of dsRNA targeting these genes for controlling *T. castaneum* populations.

It may be argued that the lack of any effects in bees is a consequence of instability of the dsRNA in the sucrose diet. Stability studies of dsRNA provided proof that the dsRNA of the target genes was not degraded significantly during 48 h. Our results agree with those of Li *et al*.,^80^ which indicated that three dsRNAs targeting different sites within a gene encoding *vacuolar ATP synthase subunit E* in *Nilaparvata lugens* were found to be stable in 0.1 g mL^–1^ sucrose solution at 22 h. The *Ap_ST1* dsRNAs (sugar transporter gene) from an aphid kept for a week at room temperature was not degraded, thus again demonstrating stability.^81^ However, 1 μg of *V‐ATPase subunit* dsRNA targeting *A. tumida* in 10 μL of 50% sucrose solution after 8 h is completely degraded.^5^ These contrasting results demonstrate the importance of evaluating the stability of specific dsRNAs under the appropriate conditions.

In conclusion, this study has shown the selective knockdown of two types of potassium ion channels in *T. castaneum*, resulting in significant mortality in the different life stages of this insect pest. Importantly, no deleterious non‐target effects were observed in honeybee, in terms of either survival or compromised immunity. It represents the first ‘proof of concept’ that targeting potassium ion channel genes by RNAi may provide a novel approach to the control of insect pests.

## Supporting information


**Figure S1.** Output from BLAST alignment tool showing sequences (a) 91% homology between plasmid insert (Query) and *SK* (sbjct); (b) 95% homology between plasmid insert (Query) and *SH* (sbjct). Line between nucleotides indicates sequence homology, whereas the absence of a line indicates a sequence change at that nucleotide.
**Figure S2.** Homology of SH and SK dsRNA fragments to known voltage‐gated potassium channel (SH) the small conductance calcium‐activated potassium channel (SK) sequences. S2a and S2b ClustalW2 alignments (https://www.ebi.ac.uk/Tools/msa/mafft/) of dsRNA template versus *T. castaneum* and *A. melifera*. S2c and S2d Neighbor‐joining tree analyses of dsRNA fragments to know sequences across insect orders. Tree constructed using Muscle aligned data with maximum composite nucleotide substitution and 1000 Bootstrap (MEGAX)
