# Supplementary material for: Targeting the potassium ion channel genes SK and SH as a novel approach for control of insect pests: efficacy and biosafety
Source: Pest Manag Sci. 2019 Jul 29;75(9):2505–16. doi: 10.1002/ps.5516 (PMC6771844; doi:10.1002/ps.5516)
Supplement: Supplementary file 1 — Figure S1. Output from BLAST alignment tool showing sequences (a) 91% homology between plasmid insert (Query) and SK (sbjct); (b) 95% homology between plasmid insert (Query) and SH (sbjct). Line between nucleotides indicates sequence homology, whereas the absence of a line indicates a sequence change at that nucleotide. Figure S2. Homology of SH and SK dsRNA fragments to known voltage‐gated potassium channel (SH) the small conductance calcium‐activated potassium channel (SK) sequences. S2a and S2b ClustalW2 alignments (https://www.ebi.ac.uk/Tools/msa/mafft/) of dsRNA template versus T. castaneum and A. melifera. S2c and S2d Neighbor‐joining tree analyses of dsRNA fragments to know sequences across insect orders. Tree constructed using Muscle aligned data with maximum composite nucleotide substitution and 1000 Bootstrap (MEGAX) [file PS-75-2505-s001.docx]

**Supplementary Figures**

Fig S1. Output from BLAST alignment tool showing sequences (a) 91% homology between plasmid insert (Query) and *SK* (sbjct); (b) 95% homology between plasmid insert (Query) and *SH* (sbjct). Line between nucleotides indicates sequence homology, whereas the absence of a line indicates a sequence change at that nucleotide.

**a**

**b**

**c**

**d**

Figure S2 Homology of SH and SK dsRNA fragments to known voltage-gated potassium channel (SH) the small conductance calcium-activated potassium channel (SK) sequences. S2a and S2b ClustalW2 alignments (https://www.ebi.ac.uk/Tools/msa/mafft/) of dsRNA template versus *T. castaneum* and *A. melifera*. S2c and S2d Neighbor-joining tree analyses of dsRNA fragments to know sequences across insect orders. Tree constructed using Muscle aligned data with maximum composite nucleotide substitution and 1000 Bootstrap (MEGAX)
